# Selective Growth of MAPbBr_3_ Rounded Microcrystals on Micro-Patterned Single-Layer Graphene Oxide/Graphene Platforms with Enhanced Photo-Stability

**DOI:** 10.3390/nano13182513

**Published:** 2023-09-08

**Authors:** Javier Bartolomé, María Vila, Carlos Redondo-Obispo, Alicia de Andrés, Carmen Coya

**Affiliations:** 1 Escuela de Ingeniería de Fuenlabrada, Universidad Rey Juan Carlos, 28933 Madrid, Spain; javier.bartolome@urjc.es (J.B.); maria.vila@urjc.es (M.V.); 2 Instituto de Ciencia de Materiales de Madrid, Consejo Superior de Investigaciones Científicas, 28049 Madrid, Spain; cd.redondo@csic.es

**Keywords:** hybrid perovskites, graphene, graphene oxide, solution-processed, stability, local anodic oxidation (LAO), AFM, X-ray diffraction, photoluminescence, excitons

## Abstract

The synergistic combination of hybrid perovskites with graphene-related materials is leading to optoelectronic devices with enhanced performance and stability. Still, taking advantage of the solution processing of perovskite onto graphene is especially challenging. Here, MAPbBr_3_ perovskite is grown on single-layer graphene/graphene oxide (Gr/GO) patterns with 120 µm periodicity using a solution-processed method. MAPbBr_3_ rounded crystals are formed with sizes ranging from nanometers to microns, either forming continuous films or dispersed particles. A detailed morphological and structural study reveals a fully oriented perovskite and very different growth habits on the Gr/GO micro-patterns, which we relate to the substrate characteristics and the nucleation rate. A simple method for controlling the nucleation rate is proposed based on the concentration of the precursor solution and the number of deposited perovskite layers. The photoluminescence is analyzed in terms of the crystal size, strain, and structural changes observed. Notably, the growth on top of Gr/GO leads to a huge photostability of the MAPbBr_3_ compared with that on glass. Especially outstanding is that of the microcrystals, which endure light densities as high as 130 kW/cm^2^. These results allow for anticipating the design of integrated nanostructures and nanoengineered devices by growing high-stability perovskite directly on Gr/GO substrates.

## 1. Introduction

Hybrid metal halide perovskites (PVKs) have demonstrated outstanding properties that have led to breakthroughs in different optoelectronic devices, such as solar cells, photodetectors, and light-emitting devices [1], with the great advantage of using low-temperature and cost-effective fabrication methods. PVKs exhibit high absorption coefficients [2], sharp absorption edges [3], a long charge carrier diffusion length [4], low exciton binding energy [5], and a tuneable band gap by chemical modification in the ideal range for silicon-based tandem devices (1.5–1.8 eV), for highly efficient homo- or hetero-multi-junctions [6]. Thus, they combine the advantages of both organic (low cost, solution-processable, and flexible) and inorganic semiconductors (high performance and high electrical conductivity). Focusing on solution synthesis methods, several strategies have reported high-quality perovskite thin films, such as sequential deposition [7], spray coating [8], the blade method [9], or the so-called solvent engineering method [10], in which different Lewis base/acid solvents or additives are used in the precursor solution to control the final morphology of the layer, as hybrid perovskites tend to grow forming grains and crystal domains due to their ionic character [10]. Actually, PVKs are the best solution-processed materials in simple layered structured devices for photovoltaic applications, where the PVK is grown directly on the lower electrode, exhibiting the highest efficiencies compared with vapour-based solar cells and 10 cm^2^ modules [11]. Interestingly, perovskite films prepared by spin-coating exhibit a trend towards the formation of highly textured/oriented films, which have been proved to be the most efficient and stable perovskite solar cells [12]. However, despite its non-epitaxial growth, it has been observed in methylammonium lead iodide (CH_3_NH_3_PbI_3_, MAPbI_3_) thin films how the nature of the underlying substrate can influence not only the preferential orientation degree and the crystal grain size, but also the lattice parameters and even the stability of the layer under ambient conditions and visible light irradiation [13].

The growth of the perovskite layers directly on graphene (Gr) or its derivatives, i.e., graphene-related materials (GRMs), could give rise to a plethora of novel applications. The ability of Gr to protect against humidity [12,14] has a fundamental influence on improving the stability of the device, which is one of the main concerns to solve in hybrid perovskite-based devices [15,16,17]. In addition, the high flexibility and optical transparency properties of Gr [18] promise that the integration of hybrid perovskite layers into graphene could lead to potential breakthroughs, for example, in tandem solar cells [19]. Also, GRMs, such as graphene oxide (GO), reduced GO (rGO), Gr quantum dots (GQDs), and other derivatives, have exhibited an effect on the control of the nucleation and growth of perovskite sheets when used as additives in the charge transport layers or the active layer due to their different functional groups [20,21,22,23]. GO features sp^3^-hybridized carbons covalently bonded to oxygen-containing functional groups [24]. These functional groups make GO hydrophilic [25], and are Lewis bases; so, they can interact with perovskite precursors and act as nucleation and crystallization centres during film growth, thus allowing for improved crystallinity and morphology of thin films, and passivating positively charged defects in the crystal structure [26]. However, there are scarce studies that consider the perovskite directly on top of Gr or GO single layers in spite of their envisioned advantages. Volonakis et al. [14] reported, by first principles calculations, that electron–hole separation and electron extraction are more efficient at the Gr or GRM–perovskite interfaces, reducing the electron–hole recombination and improving the stability of perovskite solar cells due to the formation of an interfacial dipole. In particular, GO offers a tuneable interfacial energy-level alignment depending on its oxidation grade [27], and the extraction of either holes or electrons can also be favoured. Based on these results, GO seems promising for its utilization in perovskite solar cell devices. Moreover, since GO is more reactive (due to its functional groups), it may offer more nucleation sites for perovskite growth.

However, the growth of hybrid perovskite layers by solution-processed methods directly on graphene single-layer substrates is very challenging. Its hydrophobicity is also responsible for the poor surface coverage of the perovskite layers spin-coated on it. Few studies have been reported describing the direct growth of PKVs on graphene. For instance, the selective growth by the chemical vapour deposition (CVD) of methylammonium lead bromide (MAPbBr_3_) cubic-shaped micro-crystallites on graphene was described by Liu et al. [28]. Also, Niu et al. [29] reported the growth of hexagonal MAPbI_3_ crystals in two steps: firstly, PbI_2_ nanoplatelets were obtained by CVD, and secondly, they were converted into MAPbI_3_ perovskite by reacting with CH_3_NH_3_I (MAI) under vacuum conditions. The final morphologies, sizes, and crystal orientation were different and are strongly dependent on the presence of graphene and, especially in the first case, it has been observed that graphene edges are acting as nucleation centres.

In this work, we demonstrate, using a solution-processed method, the growth of MAPbBr_3_ perovskite on single-layer GO/Gr 2D patterns. Millimetre-sized arrays of GO disks of 80 µm in diameter were formed on graphene single-layers on quartz (Gr/quartz), using a unique in-house-developed system for the local anodic oxidation (LAO) [30,31] of Gr. The growth habits were studied by scanning electron microscopy (SEM), atomic force microscopy (AFM), and X-ray diffraction (XRD) to extract morphological and structural information that allows us to present a simple model for controlling the nucleation rate in terms of the initial concentration, number of layers, and the Gr/GO substrate characteristics. Depending on these conditions, we observed rounded crystals of sizes ranging from nanometres to microns, either forming a continuous film or dispersed particles. The photo-stability and emission properties of the MAPbBr_3_ are also studied in terms of the crystal size and its strain. All this information is necessary and serves as an incentive to develop novel applications and optoelectronic devices with enhanced stability.

## 2. Materials and Methods

### 2.1. Graphene Oxide/Graphene (GO/Gr) 2D Platforms Preparation

**Figure 1:**
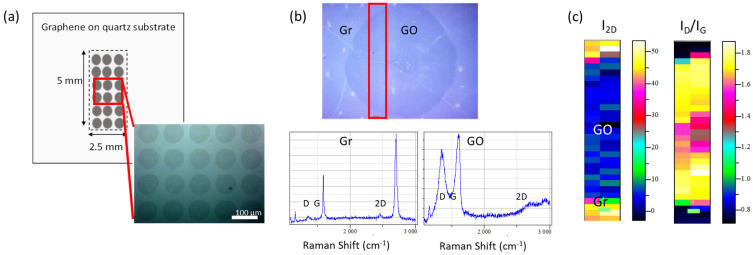
(**a**) Geometrical scheme of the 2D GO/Gr platforms and optical micrograph of the GO regions with pitch size of 120 µm and GO diameter of 80–90 µm. (**b**) Raman spectra inside and outside the GO circle, respectively, and (**c**) Raman maps of I_2D_ and I_D_/I_G_ intensity ratio for the red rectangle showing the GO and Gr.

Local Anodic Oxidation (LAO) in contact mode performed on large areas of graphene substrates was carried out with a previously reported method [30,31,32] using a home-made instrument (see Figure S1 of the Supporting Information for details). CVD single-layer graphene substrates of 1 cm^2^ size transferred onto quartz by Graphenea (San Sebastián, Spain) were used without further treatment. GO circular spots were performed every 120 µm at −40 V and 94% nominal relative humidity, covering a surface of 2.5 × 5 mm^2^ size, or 2.5 × 2.5 mm^2^ (Figure 1a). These conditions allow the formation of GO circles of 80–90 µm in diameter (Figure 1) with an oxidation degree of approximately 30%, as previously obtained from the I_D_/I_G_ intensity ratio, FWHM of G and D bands’ relationship and X-ray Photoelectron Spectroscopy (XPS) measurements in ref. [31]. Figure 1b shows the Raman spectra measured inside (GO) and outside (Gr) of the GO circle showing the D, G and 2D peaks with the characteristics of single-layer graphene and graphene oxide [33], respectively. The graphene spectrum exhibits the first-order graphene G mode at 1580 cm^−1^ and the second-order 2D mode at 2700 cm^−1^, whose shapes and relative intensities indicate a high-quality monolayer. Graphene oxide Raman spectrum shows a broad and intense defect-related D peak (≈1350 cm^−1^), a broadened graphene G peak [34], and especially, the drastic decline of the 2D band. The oxidation homogeneity in the functionalized area is shown in Figure 1c through the Raman maps of I_2D_ (high intensity reveals single-layer graphene) and I_D_/I_G_ intensity ratio (high ratio corresponds to high defect density, i.e., GO).

### 2.2. MAPbBr_3_ Synthesis on GO/Gr 2D Platforms

All materials were used as received. MAPbBr_3_ is synthetized by a stoichiometric mixture of bromide precursors, methylammonium bromide, MABr (98%), and lead bromide, PbBr_2_ (99.999%), both from Aldrich (St. Louis, MO, USA). The anhydrous solvents used were dimethyl sulfoxide (DMSO, >99.8%, Alfa Aesar, Haverhill, MA, USA) and dimethylformamide (DMF, 99.8%, Aldrich, St. Louis, MO, USA) for perovskite solution, and toluene (99.8%, Alfa Aesar, Haverhill, MA, USA) as antisolvent (AS) when needed. Two precursor solutions of MAPbBr_3_, at concentrations of either 10% or 20% wt. were prepared by dissolving PbBr_2_ in DMF:DMSO (4:1, *v*/*v*), followed by the addition of MABr to the PbBr_2_ solution. The final perovskite solution was filtered through a 0.22 µm hydrophobic polytetrafluoroethylene (PTFE) syringe filter. The solution and the substrates were heated at 60 °C before the deposition process. All samples were prepared by depositing one or two layers of perovskite by spin coating with antisolvent crystallization method at 1000 rpm for 40 s (Figure S2 in the Supporting Information). We have previous evidence that at higher speeds, no deposited layer remains on graphene. AS is added 10 s after the start, in a volume ratio three times that of the perovskite solution. An intermediate (third) layer was also deposited by vertical drop coating between the two spin-coated layers on one of the samples, without the use of any AS. The films were cured after each layer was deposited at 70 °C for 10 min on a hot plate, except for the sample with three layers, which was cured only after the last layer was deposited. For comparison purposes, single-layer thin films were also deposited on microscope glass slides from Labbox (Barcelona, Spain; silica with additives as sodium carbonate and calcium carbonate) with precursor solution concentrations of 15, 20, 25 and 40% wt., at 10 s 1000 rpms and 40 s 4000 rpms, with AS added 10 s after starting the first step and a final curing treatment at 100 °C for 10 min on a hot plate. The entire process was carried out in the inert N_2_ environment of a glove box. All the characterization and subsequent measurements were carried out at room conditions, without any type of encapsulation. The samples are named using a generic labelling in the form of X%NL, where X is the concentration of the PKV precursor solution and *N* the number of layers. The details of the growth process of each sample can be seen in Table S1 in the Supporting Information.

### 2.3. Structural and Morphological Characterization

A commercial Nano-Observer AFM (Scientec, Les Ulis, France) operating in ambient conditions was used to study the topography of the samples, working in tapping mode. Image analysis was performed with the free software Gwydion [35]. When needed, the calibration of the substrate level was carried out on images obtained on graphene regions with pits, under the assumption that these pits go through the entire thickness of the film down to the substrate. Topographic SEM images were acquired with a FEI/Philips (Hillsboro, OR, USA) XL30 ESEM microscope. Secondary electron (SE) and backscattered electron (BSE) signals were used for the electron micrographs, showing essentially the same information. However, since the BSE signal is more insensitive to sample charging effects (caused by the low conductivity of the films), it was preferred over the SE signal for imaging most of the samples. X-ray powder diffraction (XRD) profiles of MAPbBr_3_ on GO/Gr substrates were registered employing a Bruker (Billerica, MA, USA) D8 Advance diffractometer using Cu Kα radiation over a 2θ range between 8° and 65° with a step size of 0.02°. Peak fitting to Lorentz peak profiles was performed after filtering the Kα_2_ component from the data. Profilometry measurements were made using an Alpha Step 200 (KLA-Tencor Instruments, Milpitas, CA, USA).

### 2.4. Absorption and Photoluminescence Measurements

UV-VIS absorption spectra of the thin films were obtained using a UV-VIS-NIR spectrophotometer (Varian, Cary 500, Paso Alto, CA, USA) in the wavelength range of 300 to 900 nm.

Steady-state photoluminescence emission (PL) of the films on glass was carried out using an AVANTES (Apeldoorn, The Netherlands) high-sensitivity fibre-optic spectrometer, model AvaSpec-HERO (100 mm optical bench, NA of 0.13), a Peltier-cooled, back-thinned detector and a 30 mm integrating sphere (250–2500 nm) AvaSphere-30-REFL. The excitation wavelength was 360 nm (10 mW) from a solid-state fibre-coupled laser (CNILASER, Changchun, China). Micro-photoluminescence (µ-PL) spectra of PVK on graphene were measured using a 488 nm excitation wavelength of an Ar+ laser in backscattering geometry with an Olympus (Tokyo, Japan) microscope and a Horiba (Kyoto, Japan) iHR-320 monochromator coupled to a Peltier-cooled Synapse CCD. The light was collected from spots <1, 5, and 25 μm in diameter (corresponding to ×100, ×20 and ×4 objectives). Different neutral optical filters (up to OD 4) were used to reduce the incident power on the samples; so, the resulting power density can be varied, depending on the objective, from 400 kW/cm^2^ to 0.6 W/cm^2^). The best resolution, obtained with a high N.A. ×100 objective, is ~0.8 µm using a 488 nm excitation wavelength. µ-PL images are obtained with steps of 1 or 5 µm. Each point in the µ-PL images corresponds to the integrated intensity in the 500 to 600 nm range of the spectrum, corresponding to each position in the sample. For PL time evolution, the full spectra are obtained at different times and, either the succession of the spectra (as a 2D image) or the integrated intensity of each spectrum in the range 500 to 600 nm are plotted as a function of time. All measurements were carried out in ambient conditions (40–50% of relative humidity (RH)).

## 3. Results

### 3.1. Morphological and Structural Characterization

**Figure 2:**
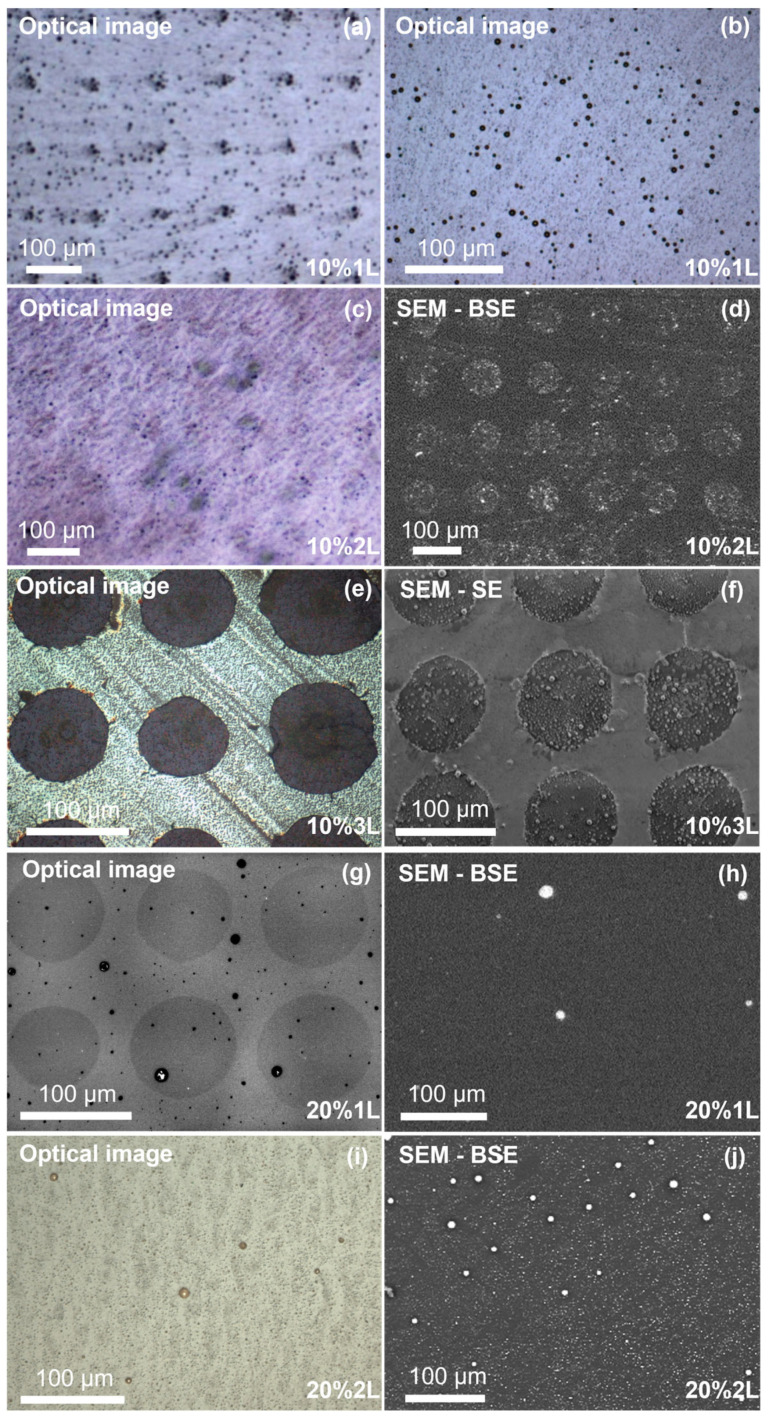
Surface morphology of MAPbBr_3_ films deposited on Gr/GO micropatterns. (**a**,**b**) Sample 10%1L, comparison between optical images obtained on top of and outside the micropattern. (**c**,**d**), (**e**,**f**), (**g**,**h**), (**i**,**j**) are comparisons between optical and SEM images recorded on top of the micropattern of samples 10%2L, 10%3L, 20%1L and 20%2L, respectively.

To explore the different growth habits of the PKV on single-layer Gr/GO micropatterned substrates, five different samples were synthesized, namely samples 10%1L, 10%2L, 10%3L, 20%1L and 20%2L, according to the labelling explained previously (Table S1 of the Supporting Information). Figure 2 shows optical and SEM micrographs of the PKV deposited on top of the micropatterned substrates. Sample 10%1L shows the appearance of round crystallites scattered on its surface, presenting a wide distribution of lateral sizes (diameters) ranging from few tens of nm up to 1–2 µm. Outside the micropatterned region, the crystals seem to be randomly distributed; however, inside the GO micropattern, particularly big crystallites (average diameter of ~4.5 µm) tend to cluster at the centre of the GO spots, forming a very regular square array that mimics the GO/Gr pattern (Figure 2a,b).

A closer inspection of the GO spots shows a slightly brighter contrast in the optical images compared to the surrounding Gr areas, suggesting that either less material is deposited on top of the GO spots, or it is concentrated on the large crystallites in the centre (see Supporting Information Figure S3). The addition of a second PKV layer (sample 10%2L) increases both the concentration and size of the round crystallites dispersed throughout the sample surface, as evidenced by the AFM images on the Gr regions, showing a denser dispersion of small crystallites of few hundreds of nm (see Figure S4 in the Supporting Information). However, this effect is clearly enhanced in the GO spots (SEM images, Figure 2c,d), with crystallites of up to a few µm in diameter clustered inside the spots. In general, the aspect ratio (diameter/height) of the crystallites is around 10, irrespective of the sample or region observed.

**Figure 3:**
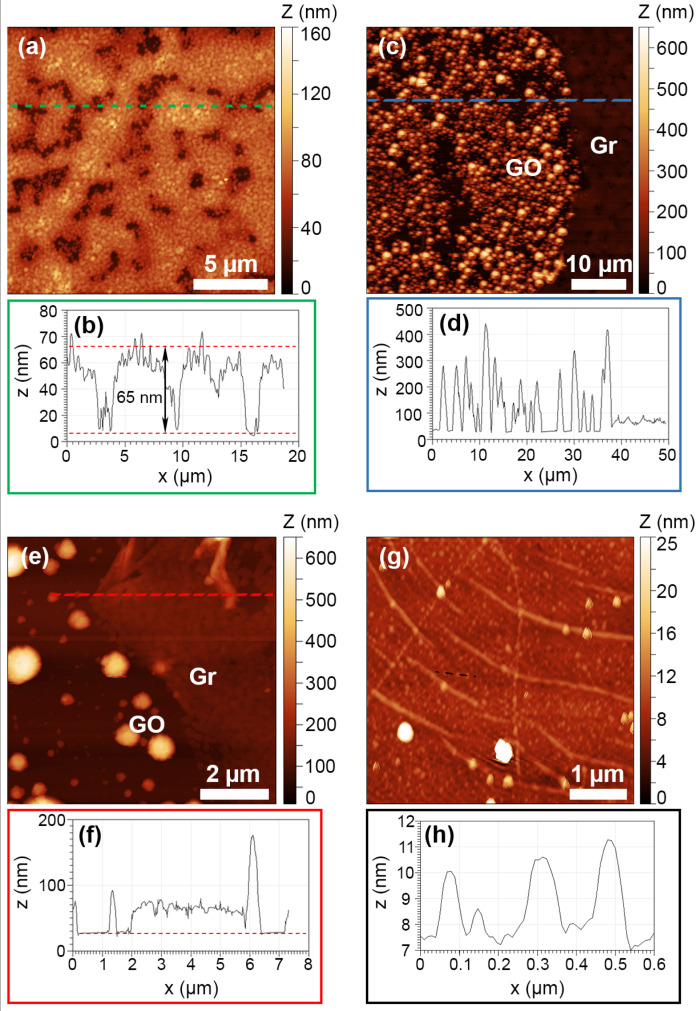
AFM images and corresponding line profiles across the indicated dashed lines of (**a**,**b**) sample 10%3L over the Gr region, (**c**,**d**) overview of a GO spot on the same sample, and (**e**,**f**) detail of the boundary between GO and Gr regions on (**c**). Dotted line in (**f**) is used as guide for the eye. (**g**,**h**) Surface of the sample 20%1L, showing the presence of graphene wrinkles. The image was filtered using a 2D FFT filter to eliminate a 50 Hz noise from the power grid. The line profile shows the typical size of four example crystallites.

Depositing three layers in a row without any intermediate curing step (sample 10%3L) leads to a great increase in the crystallite density on top of the GO spots, while leaving the surrounding Gr areas relatively free of crystallites (Figure 2e,f). Figure 3a shows an AFM image of the topography of the sample over the Gr region, revealing that it is actually covered by a relatively continuous PKV layer formed by coalesced grains with an average diameter of 180 nm and an estimated layer thickness of 65 nm (Figure 3b).

The surface reveals a slightly corrugated morphology, which is typical of antisolvent methods and has previously been related to in-plane compressive stress that is released by the formation of the observed wrinkles [36]. In comparison, GO spots show very rough surfaces due to the presence of the crystallites with slightly narrower size dispersions compared to sample 10%1L. Large crystallites are just on the order of 1–2 µm in diameter, while small crystallites are in the range of a few hundred nm in diameter (Figure 3c,d). Hence, depositing several layers without intermediate curing steps has the effect of increasing the homogeneity and density of the PKV particles on both the Gr and the GO regions, leading to a continuous film on the Gr region. Interestingly, no PKV is deposited in between the crystallites on the GO spots (Figure 3e,f), with all the material concentrated in the crystallites themselves. Despite this, measurements of the total deposited volume per unit area show that there is actually more material deposited on the GO spots, with an average thickness (integrated height divided by total area) of approximately 145 nm, while on the Gr areas the average thickness is ~65 nm. Therefore, GO promotes the deposition of MAPbBr_3_ PKV over Gr by solution process routes under low precursor concentration, favouring the formation of micro-sized crystals.

Increasing the precursor concentration up to 20% completely changes the appearance of the samples. Figure 2g,h show relatively homogeneous surfaces for sample 20%1L, with GO spots only visible in the optical micrographs, indicating that no topography changes are visible between Gr or GO regions. The contrast observed on the optical micrograph is most likely produced by the GO itself, as explained below. Some large crystallites are scattered over the surface of the sample, with no apparent order or correlation with the GO spots. Detailed images of the sample surface obtained by AFM (Figure 3g,h) reveal that it is actually covered by disconnected nanoparticles with diameters in the range of 100 to 150 nm, and heights of 2 to 3 nm. The space between the particles seems to be empty of any PKV, similar to the case of the GO spots in sample 10%3L, as suggested by the fact that graphene wrinkles are clearly visible in the image, and the lack of any other features in the background. Interestingly, the appearance of larger crystallites (several hundreds of nm in diameter) seems to be associated with the presence of graphene wrinkles, suggesting that they may act as preferential nucleation points in graphene, possibly due to their higher strain and/or concentration of defects, in agreement with observations reported in previous works on MAPbBr_3_ crystals grown on graphene by CVD [20]. Depositing two layers with an intermediate curing step drastically increases the density and size distribution of the crystallites (sample 20%2L, Figure 2i,j); however, similar to the case of sample 20%1L, the crystallites seem to be uniformly distributed, with no visible influence from the presence of GO spots.

**Figure 4:**
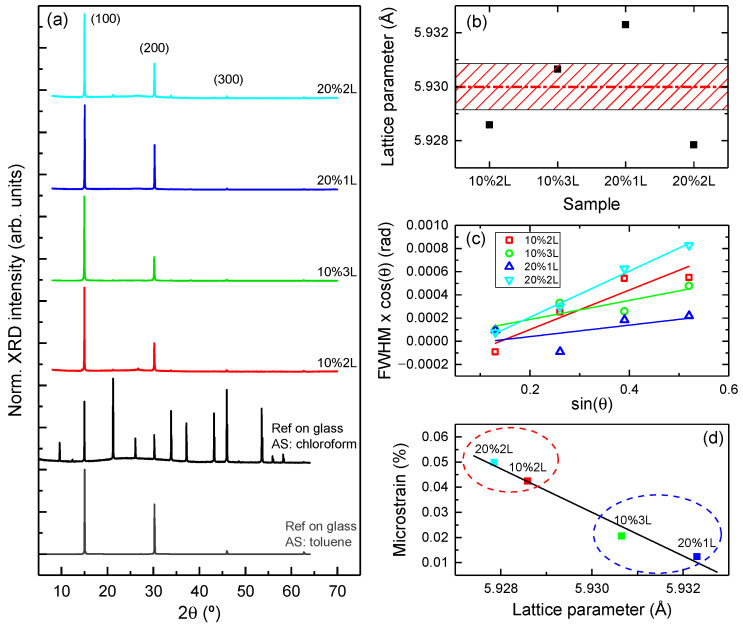
(**a**) Representative XRD patterns obtained on samples 10%2L, 10%3L, 20%1L and 20%2L, as well as on two reference samples deposited on glass using a similar procedure but employing either toluene or chloroform as AS. (**b**) Calculated lattice parameters. The red dashed line shows the average value for the samples on GO/Gr and the reference samples on glass, the shadowed area shows the standard deviation for the samples on glass. (**c**) Williamson–Hall plots. (**d**) Samples microstrain as a function of their lattice parameter.

XRD patterns of the obtained samples (Figure 4a) confirm that the deposited layers consist of MAPbBr_3_ PKV with a cubic structure ($Pm\underline{3}m\;space\;group$) and no other phases are present within the detection limit of the technique. Some spurious reflections are visible in some samples, coming either from the quartz substrate or from the silver paste used to electrically ground the substrates during the patterning of GO on graphene. The samples are almost fully textured, with the (100) crystal planes oriented parallel to their surface. This is an unexpected result considering the rounded shape of the crystallites, which would suggest a more random orientation. XRD patterns obtained from two films deposited on glass using two different antisolvent compounds, either toluene or chloroform, are also shown in Figure 4a for comparison. Toluene, when used as antisolvent, is known to yield highly textured films, while chloroform induces almost polycrystalline films. It is clear from the figure that all samples are very similar to the reference with toluene in terms of film orientation. Plotting the XRD patterns on a logarithmic scale shows the presence of very weak reflections from other crystal planes (not shown), particularly in sample 10%3L, which has the highest XRD intensity, revealing a certain (almost negligible) polycrystalline fraction within the samples. The rocking curve of the (100) peak for the 10%3L sample (Figure S5 in the Supporting Information) shows a width around 4°, which reveals a quite narrow orientation of the (100) crystallites.

Fitting the peaks of the systematic (n00) reflections up to the (400) contribution allowed the determination of the lattice parameter, shown in Figure 4b. The average value of 5.930 Å is the same as that of the films deposited on glass at different concentrations (red dashed line in Figure 4b) and is consistent with the literature [37,38]. However, when examined independently, it is apparent that those samples with two layers and an intermediate curing step (10%2L and 20%2L) have a similar but smaller lattice parameter (Figure 4b). To further investigate this difference, the microstrain of the samples was determined using the Williamson–Hall procedure, where the full width at half maximum, $FWHM_{\left(hkl\right)},$ of each hkl peak times $\cos\theta_{hkl}$ is plotted against $\sin\theta_{hkl}$, according to the following relationship:

$$FWHM_{\left(hkl\right)}=\frac{K\lambda }{D\cos\theta_{hkl}\;}+4\varepsilon \frac{\sin\theta_{hkl}\;}{\cos\theta_{hkl}}$$

where λ is the wavelength of the X-ray, K is the shape factor (0.9), D is the crystal size, ε is the microstrain and $\theta_{hkl}$ is the angle of the hkl reflection. Thus, Williamson–Hall plots separate the contributions of crystal size and microstrain to the XRD peak broadening into two independent parameters, the intercept and slope of a linear relation, respectively [39]. From the Williamson–Hall plots of Figure 4c, it is evident that the four samples can be organized into two groups with two different slopes: one comprised by the samples with two layers and an intermediate curing step (10%2L and 20%2L), and the other by the samples with no intermediate curing steps (10%3L and 20%1L). The same procedure for the reference samples reveals that the films on glass are not inhomogeneously strained (negligible slope in the Williamson–Hall plots). The microstrain calculated for samples on Gr/GO (Figure 4d) shows a clear linear correlation with the obtained lattice parameters, with samples with the highest microstrain also presenting the smallest lattice parameter. This suggests that the deposition of a second layer after a curing step induces an inhomogeneous compressive strain in the sample, which increases the microstrain and reduces its overall lattice parameter. Conversely, if no intermediate curing steps are used, the deposition of successive layers does not have a significant impact on the lattice parameter and only low microstrain is generated. One possible way to interpret this result is as follows: when no curing is performed between layer depositions, then depositing the next layer leads to the partial dissolution and regrowth of already formed crystals, avoiding defect accumulation and the associated strain. On the other hand, when the samples are cured before the next layer is deposited, then the partial dissolution of the crystals is prevented or at least hindered, and a stack of layers is directly formed one on top of the other. In this case, defects easily accumulate at the interface between layers, leading to increased (in this case, compressive) microstrain.

### 3.2. Growth Model

Based on the results of the previous section, a growth model for perovskite on GO/Gr micropatterned substrates is proposed. Graphene is known to be a very stable, impermeable and hydrophobic material, which hinders the formation of PVK nuclei, reducing its nucleation rate. On the other hand, GO has different functional groups and defects that may actually act a preferential nucleation sites for the PKV, promoting the nucleation rate at these sites.

**Figure 5:**
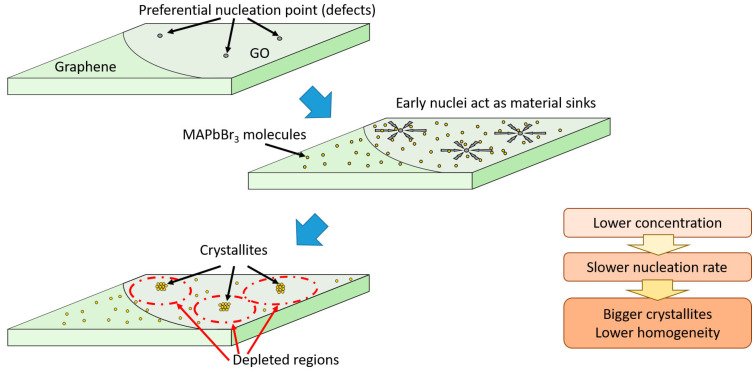
Schematic for the growth MAPbBr_3_ PKV on the GO/Gr micropatterned substrates.

At low concentrations of precursor solution, the nucleation rate is generally slow and PKV initially only forms at these preferential nucleation sites. Once the nuclei are formed, they start growing quickly, depleting the surrounding medium of PKV solute and preventing the formation of further nuclei in their vicinity (Figure 5). This is the reason why sample 10%1L shows such large crystallites at the centre of the GO spots while the rest of the spot has less material compared to the surrounding Gr areas. Graphene usually becomes destroyed at the point of contact between the tip of the anodic oxidation system and the substrate (the centre of the GO spots) due to the initial high currents (spark), creating an area of few microns where no graphene or highly defective graphene is present [30,31]. This point is an excellent nucleation site for the PKV, leading to the rapid formation of big crystallites at the centre of the GO spots. This is supported by theoretical calculations associating nucleation sites with higher electron densities around oxygen atoms (present in these defects and in GO) of Lewis bases [40]. Conversely, in the Gr regions, the nucleation rate is highly diminished and very few preferential nucleation points are present, leading to the growth of more homogeneous, smaller and more densely packed crystallites, sprinkled with some bigger crystallites where these rare preferential nucleation points are present, such as the graphene wrinkles (Figure 3d). Adding subsequent layers without a curing step strongly dissolves the small crystallites in the Gr regions, locally increasing the concentration of the solution and restarting the nucleation process. This concentration increase enhances the nucleation rate and hence more crystallites are formed without significantly increasing their overall size, leading to a continuous film over the Gr regions observed in sample 10%3L. Dissolution and regrowth of the crystallites also takes place at the GO spots; however, in this case, since there is still a high density of preferential nucleation sites, more nuclei are rapidly formed, increasing the number of crystallites and homogenising their size at the cost of almost no change in size for the largest crystallites. Using an intermediate curing step reduces the amount of material from the previous layer that dissolves, and thus the nucleation points still available on the Gr and GO regions have to compete with the undissolved crystals already formed. These crystals rapidly consume the precursor solution, hindering the formation of new nuclei. Thus, the overall effect is a limited increase in crystallite density, compared to the case where no curing was performed, and a noticeable increase in crystallite size.

At higher precursor concentrations, the nucleation rate is strongly increased all over the samples, and thus the difference in nucleation rate between the preferential nucleation sites on the GO spots, and the rest of the sample, is considerably reduced. Hence, more crystals are nucleated simultaneously, leading to a homogeneous distribution of crystallites with no clear differences between Gr and GO regions, as observed in sample 20%1L. Depositing a second layer after curing the sample leads to the growth of the crystallites already present, which consume the precursor, hindering the nucleation of new crystallites, and leading to larger crystallite sizes, without yet differentiating between the Gr and GO regions.

### 3.3. Photoluminescence Properties

**Figure 6:**
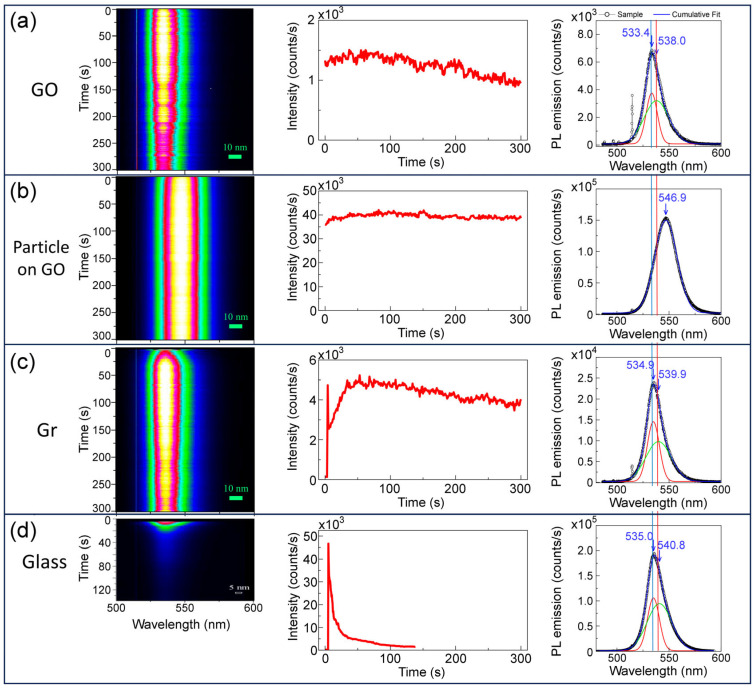
Evolution of PL with irradiation time under 16 W/cm^2^ at 488 nm for the 10%1L sample at (**a**) GO region, (**b**) large particle on GO and (**c**) on graphene; (**d**) evolution for a film deposited on glass. From left to right: 2D image of the spectra with time, integrated intensity (500–600 nm) with time and spectrum at the initial stage (black line with open circles) with its fitting to Gaussian contributions (red and green lines: individual components, blue line: total fit).

In addition to the peculiarities of the morphology of the MAPbBr_3_ perovskite formed on graphene and GO, which differs significantly from that on glass or PEDOT:PSS [13], the emission characteristics are also modified to a different extent. The most remarkable aspect is the huge improvement of stability under high visible light irradiation density. Figure 6 shows the evolution of the photoluminescence (PL) over time for different spots in sample 10%1L and compares it with that of a film deposited on glass. Three representative spots in 10%1L are shown: in GO with no apparent particles (a), in a 5 µm particle on GO and finally in the continuous graphene film.

Under moderate 488 nm light density (16 W/cm^2^), the PL signal for the film on glass depletes rapidly, (tens of seconds) while for sample 10%1L, on GO and Gr substrates, it is quite stable, especially for the large particle on GO which remains invariable during the measurement. The 2D images on the left (evolution of the spectra with time) clearly show the different behaviour of photostability, as well as the red shift of the PL position for the large particle on GO. On the right side of the figure, the initial spectra are shown with fits to two Gaussian peaks (only one for the large particle signal). The exceptional photostability of the perovskite on GO and graphene maintains about 70% of the PL intensity after 130 s under very high power density (130 kW/cm^2^). The large particles are even more resilient: PL is only reduced by 20% under 130 kW/cm^2^.

**Figure 7:**
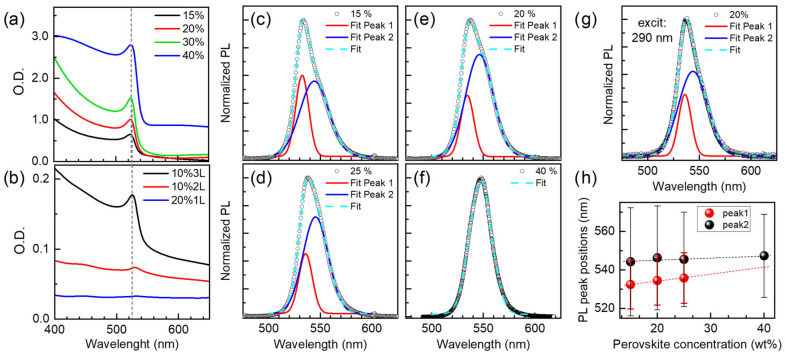
Absorbance of (**a**) the films on glass obtained with different concentrations; (**b**) the 10%1L, 10%3L and 20%1L samples on patterned Gr/GO; (**c**–**f**) PL spectra of the films on glass with the corresponding fits, (**g**) PL spectrum with 290 nm excitation; (**h**) positions of the two contributions versus concertation for the films on glass, the FWHM of the two PL peaks are represented by the error bars.

In the case of MAPbBr_3_, cm sized single crystals in different PL positions have been reported for the bulk (PL at 560 nm, 2.217 eV) and for the surface (540 nm, 2.30 eV) [41]. The differentiation was obtained comparing the PL with single-photon excitation at 400 nm (high absorption, signal from 80 nm deep) and two-photon excitation with an 800 nm laser (low absorption, bulk). The difference in PL positions is related to differently strained core and shell. However, asymmetric PL bands, with a tail in the red side, are generally reported for MAPbBr_3_. Droseros et al. have used two peaks to fit the PL signal of polycrystalline films [42], which are assigned to two different transitions, one arising from radiative recombination of free electrons (band-to-band) for the higher-energy component and the lower one due to excitons. The exciton binding energy for this compound is typically in the 30 to 100 meV range. Indeed, an excitonic absorption is reported in MAPbBr_3_ and is clearly observed in the absorption spectra of films on glass (Figure 7a) as well as in samples on Gr/GO (Figure 7b), which supports the presence of two contributions to the PL. On the other hand, the position of the PL band is reported to blue-shift for NPs below 100–50 nm [42,43]. The origin of the shift has been related either to the suppression of exciton trapping due to passivation of surface traps as NP size is reduced [42], such that only the higher energy free-carrier contribution is occurring or, in MAPbI_3_, to Pb-I distance shrinking [44].

There are therefore two different factors that determine the PL band position and shape. On the one hand, the strain, which may be different at the core of large crystals (PL at 560 nm, [32]), compared to its external shell, which has been considered compressively strained (540 nm, [32]). Similarly, nanoparticles below 50–100 nm may be further compressively strained [35], leading to the reported blue-shift in the PL spectra (525–505 nm, [33]). On the other hand, two components, related to free carriers and excitons, can present different weights depending on the size of the particles, which would result in a shift of the PL band maximum, though not necessarily of the components themselves. In the present case, 488 nm laser excitation was used and asymmetric PL bands are most frequently seen, but symmetric bands are obtained in some cases. The 488 nm excitation used is close to the absorption minimum above the MAPbBr_3_ bandgap (Figure 7a,b); so, the PL is estimated to originate from up to ~160 nm depth. Two peaks, around 534 and 545 nm, are required to fit the PL of films deposited on glass (Figure 7c–f). To discern whether the two contributions are due to core and shell emissions (since the penetration depth is double than in ref. [32], both contributions can be accessible) or to free electron and exciton transitions, a 290 nm laser excitation was used to record the PL of the films (Figure 7g). The signal originates very close to the surface due to the very high absorbance at 290 nm, but the same two components are still detected. Thus, we consider that both components are intrinsic mechanisms (free electrons and excitons). However, the substrate and the crystal size and strain seem to play a role in the variation of both PL peak positions, and in the balance between both contributions.

The morphology of the PVK formed on graphene and GO is complex and involves, in some cases, particles with size distributions at different scales (nm and micron) within the same region. Thus, as a first step, we analyse the variation of the PL band for films deposited on glass as a function of the initial solution concentration, which results in different grain size and size distribution functions. As the grain size increases from 70 nm to around 1 micron (for 15 to 40 % concentrations, Figure S6) the positions of both contributions are slightly red-shifted (lower energy) (Figure 7h). The free electron contribution is always much narrower (~13 nm) than that of the exciton (~27 nm) with a more important weight of the latter (peak widths are represented by error bars in Figure 7h). The exciton energy (as the difference of both transition energies) is slightly reduced (from 51 to 30 meV) and, for the film with larger grains, only one contribution is detected. This may indicate that the exciton energy is too small so the peaks are too close to be resolved. With all this in mind we now discuss the data from the samples deposited on graphene and GO.

**Figure 8:**
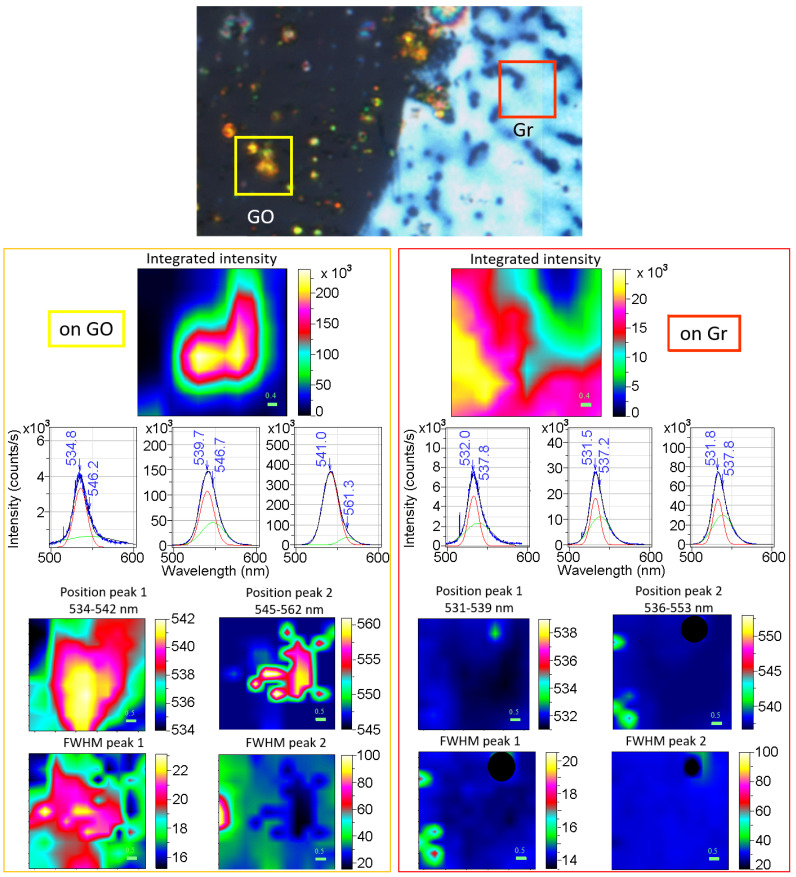
An optical image of sample 10%3L shows a region close to the edge of a GO-patterned circle (upper part). The details of the PL of the perovskite on GO (yellow square area) and Gr (red square area) are presented on left and right sides, respectively. For each area, maps of the integrated PL intensity (500–600 nm range) and maps of the positions and widths (FWHM) of the two fitted peaks are presented. Incorrect fitting values were obtained in a particular pixel on the maps of the Gr region; so, these values have been hidden (dark circles in the maps) in order to avoid a meaningless distortion of the maps. Representative low-, medium- and high-intensity spectra for the graphene and GO areas are shown (blue lines), with their two Gaussian fits (red and green lines). Note that the intensity scales are different.

The PL characteristics of the samples 10%1L and 10%3L are analysed using micro-photoluminescence hyperspectral data from different areas on graphene and GO. PL intensity maps, showing the integrated intensity of the whole PL band for sample 10%3L for a region on graphene (5 × 5 µm^2^ red square in the central optical image) and another on GO (yellow 5 × 5 µm^2^ square) are represented in Figure 8. Representative spectra are plotted in the figure for each region, with the fits to the two components. The positions and widths of the two peaks are presented as images (left for the perovskite on Gr and right on GO).

For the 10%3L sample on graphene, the film is continuous (65 nm thick) with narrow grain size distribution and an average in-plane size of 180 nm (Figure 3a). It, however, presents pits (dark features in the optical image in Figure 8) which are also revealed in the PL intensity map (Figure 8, top left). The inter-particle distances are small enough (Figure 3a) so that a PL signal, originating at 0.8 µm ∅ spots, does not vanish. Representative spectra with the fitted Gaussian functions are shown. The positions and widths of both components (Figure 8, left) evidence fully homogeneous characteristics (PL1 peak at 532 nm and 14.5 nm width and PL2 at 539 nm and 30 nm width). The band-to-band component is similar to that of the film on glass (15%) with the smaller grains (70 nm), but the exciton peak position (539 nm) is blue-shifted (Figure 7). Also, both components are blue-shifted compared to those of the 10%1L sample with larger grains. The diffraction analysis shows that the lattice parameter of the 10%3L sample is almost identical to that of the films on glass (Figure 4c); however, the XRD signal arises mainly from the large particles since no size-related broadening (expected for a 65 nm thick film) is detected and no information can be isolated for the thin PVK film on graphene. For the grains that form the continuous film on Gr, which have a quite high aspect ratio (around 3), and since the blue-shift has been related to size reduction, we may speculate that the relevant dimension could be the shortest (perpendicular to the surface) and that a compressive strain occurs in this direction. Such in-plane strain, morphologically revealed in the form of wrinkles, is characteristic of the AS method, as mentioned above [27]. This strain may also be at the origin of the smaller-than-expected exciton binding energy (31 meV) compared to the trend observed in film-on-glass series where binding energy depletes as grain size increases (Figure 7h).

On GO, as described previously, no film is formed. The particles present two size regimes: grains with an average size of 600 nm and about 3% above 1 µm. On the right side of Figure 8, the PL of a region on GO (yellow square) with small particles (black in the optical figure) and large, >1 micron, (bright spots) is presented. Spectra of regions of low, middle and high intensities are shown with the fitted Gaussians. The weaker PL spectra present positions and widths close to those of the 20% film on glass while, for the PL from the large grains (>1 micron), one very prominent peak at ~541 nm (wider than expected) is detected together with a small and quite narrow (<20 nm) component at 560 nm. This weak and narrow peak at 560 nm coincides well with that assigned to bulk signal from single crystals [32]. Thus, we suggest that, for these large particles, the shell and the bulk are detected and that the exciton binding energies in both cases are small and not discernible as it occurs to the film on glass with the bigger grains (40%). The PL features of 10%1L, which presents grains with two size distributions (hundreds of nm and particles > 1 µm) with different densities for graphene and GO, follow similar behaviours to those on GO for the 10%3L sample.

To conclude, in the PL spectra of polycrystalline films on glass, we observe two components in general, assigned to free carriers and excitons, which red-shift as the grain size increases from 70 nm to 1 µm. A progressive reduction of the exciton binding energy (from 50 to 30 meV) is also revealed. For larger grains (>1 µm) only one component is detected, probably due to reduced binding exciton energy. The PL of continuous perovskite films on graphene, 65 nm thick and 180 nm in diameter grains, resembles to that of the film on glass with 70 nm grains, but with a blue-shifted exciton peak and consequently, a lower-than-expected binding exciton energy. We propose that both effects are due to the strain. Isolated grain PL behaves similarly to films on glass. For the largest grains (>1 µm) on GO and graphene, a small and narrow component at high wavelength (~560 nm) may be related to the unstrained core similarly to the signal reported in large single crystals. Finally, the continuous thin perovskite layers on graphene and GO show PL intensities several orders of magnitude lower than those of the large grains, most probably due to efficient quenching in the former case.

## 4. Conclusions

Direct growth of MAPbBr_3_ perovskite on 2D Gr/GO micropatterns has been achieved using solution-processed methods and governing factors that determine its morphology and photoluminescence properties have been investigated. MAPbBr_3_ crystals, either forming a continuous film or as dispersed particles of nano-to-micro sizes are obtained, depending on the nucleation rate and the different nature of Gr and GO. The films exhibit high crystallinity and are almost fully textured, with the (100) crystalline planes oriented parallel to their surface. A simple model is proposed to explain the morphology of the obtained films, based on the concentration of the precursor solution and the density of available nucleation sites. At low precursor concentrations, the high density of preferential nucleation sites of GO promotes the rapid formation of sparse microcrystals, preventing the formation of more nuclei around them. Conversely, the low density of preferential nucleation sites in graphene forces the MAPbBr_3_ to grow more homogenously, favouring the formation of a continuous layer. The increase in the nucleation rate, by increasing the concentration of the precursor solution, diminishes the relevance of the different density of preferential nucleation points between GO and the Gr, and therefore the PVK grows more homogeneously. Depositing multiple layers with an intermediate curing step leads to the rapid overgrowth of the already existing crystallites, increasing the inhomogeneity of the samples, and inducing compressive microstrain possibly due to the accumulation of defects at the interface. However, if no curing is performed between layers, the precursor solution partially dissolves the previously deposited MAPbBr_3_ before forming the new layer, which, on the one hand, prevents the accumulation of compressive microstrain at the interface and, on the other hand, allows the formation of new nuclei on the GO region, increasing its density of crystallites, while permitting the growth of a continuous layer on the Gr region.

In most cases, two components are detected in the PL spectra that are assigned to transitions of free electrons and excitons. The peaks are red-shifted and the exciton binding energy diminishes as the size of the crystals increases from tens of nm to one micron. The wavelength changes and lower binding exciton energy compared to the films on glass are proposed to be due to the observed microstrain. Only one component in the PL is resolved for the larger grains (>1 µm), most probably caused by the small exciton binding energy. For even larger grains on GO and graphene, a small and narrow component at the same wavelength (λ= 560 nm), as the signal reported in cm sized single crystals, may be related to the unstrained core of the micron sized crystals. The PL arising from more-or-less-continuous thin perovskite layers on graphene and GO is several orders of magnitude smaller than that of large grains, probably due to an efficient quenching in the former case. But, remarkably, MAPbBr_3_ grown on the Gr/GO platforms with any morphology exhibits huge photo-stability compared to that on glass. Especially outstanding is that of the micro-crystals on GO which endure light densities as high as 130 kW/cm^2^. These results could allow the design of high-performance applications in integrated nanostructures and nano-engineered devices by growing highly stable perovskite directly on Gr/GO substrate.

## Data Availability

Raw data are available on request.

## Supplementary Materials

The following supporting information can be downloaded at https://www.mdpi.com/article/10.3390/nano13182513/s1. Figure S1: Setup for local anodic oxidation; Figure S2: Schematic of the PKV deposition; Table S1: growth conditions of the samples; Figure S3: Optical image of sample 10%1L; Figure S4: AFM image of sample 10%2L; Figure S5: Rocking curve; Figure S6: AFM images of films on glass.
