# Supplementary material for: Selective Growth of MAPbBr3 Rounded Microcrystals on Micro-Patterned Single-Layer Graphene Oxide/Graphene Platforms with Enhanced Photo-Stability
Source: Nanomaterials (Basel). 2023 Sep 8;13(18):2513. doi: 10.3390/nano13182513 (PMC10538007; doi:10.3390/nano13182513)
Supplement: Supplementary file 1 [file nanomaterials-13-02513-s001.zip › nanomaterials-2548660-supplementary.pdf]

SUPPORTING INFORMATION

Selective Growth of $MAPbBr_3$ Rounded Microcrystals on Micro-Patterned Single-Layer Graphene Oxide/Graphene Platforms with Enhanced Photo-Stability

Javier Bartolomé [1,†], María Vila [1,†], Carlos Redondo-Obispo [2], Alicia de Andrés [2,*] and Carmen Coya [1,*]

[1] Escuela de Ingeniería de Fuenlabrada, Universidad Rey Juan Carlos, 28933 Madrid, Spain.

[2] Instituto de Ciencia de Materiales de Madrid, Consejo Superior de Investigaciones Científicas, 28049 Madrid, Spain

S1. Local Oxidation method of graphene single-layer (LOC).

A home-made instrument (Figure S1a, [32]) was used for the local anodic oxidation (LAO) on graphene substrates according with a previously reported method [30,31]. Briefly, the LAO instrument is equipped with 3 electromechanical linear stages (PI PLS-85, MICOS) for movement on a micrometric scale, in which have been coupled a PI Hera XY piezo stage (XY) and a PI Lisa linear piezo-actuator (Z) (Figure SI1b). This allows a maximum travel for the XY axes reaching 100 µm with a resolution of 0.4 nm and a repeatability of ±2 nm and 38 µm for vertical direction with a resolution of 0.1 nm and a repeatability of ±3 nm. The implemented motion control allows automatized tilt correction upon previous mapping of the surface plane, so the force exerted on the sample may be kept constant during operation over large areas (> mm^2). It is included a power supply (0–100 V) and high-speed oscilloscope (1.5 GHz, 10 Gs s−1) to monitor and record the electrical response during operation. A plastic cover and internal cold vapour generator ensure stationary humidity conditions, with a computer monitored thermo-hygrometer sensor.

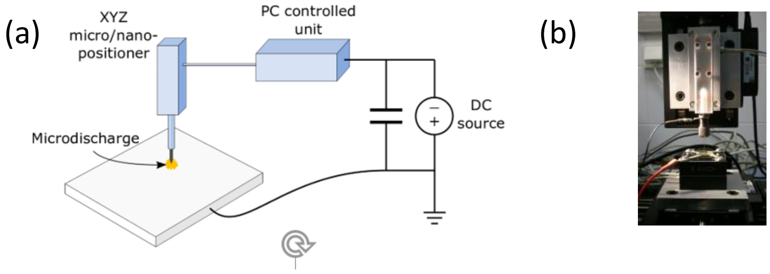

Figure S1. (**a**) Schematic setup of the local anodic oxidation (LOC) system and (**b**) detail of the 3 electromechanical linear stages (PI PLS-85, MICOS) for movement on a micrometric scale, in which have been coupled a PI Hera XY piezo stage (XY) and a PI Lisa linear piezo-actuator (Z).

$MAPbBr_3$ synthesis on GO/G 2D platforms.

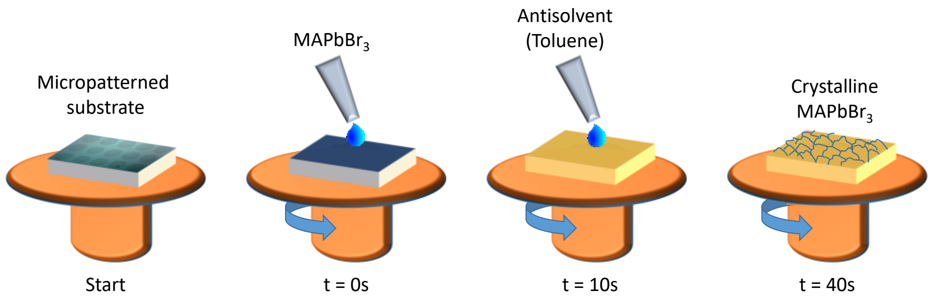

Figure S2. Schematic of the PKV deposition process by spin coating.

The details of the experimental procedure for each sample are collected in the following table

Table S1. Summary of the growth conditions of the samples studied in this work. Where omitted spin coating was used. AS stands for anti-solvent (toluene).

Sample	# layers	$MAPbBr_3$ growth			
		Step 1	Step 2	Step 3	Step 4
10%1L	1	**1000 rpms, 40 s** PKV(10%) + AS	**Curing** 3 min@70 °C		
10%2L*	2	**1000 rpms, 40 s** PKV(10%) + AS	**Curing** 3 min@70 °C	**1000 rpms, 40 s** PKV(10%) + AS	**Curing** 3 min@70 °C
10%3L	3	**1000 rpms, 40 s** PKV(10%) + AS	**Drop coating** PKV(10%)	**1000 rpms, 40 s** PKV(10%) + AS	**Curing** 3 min@70 °C
20%1L	1	**1000 rpms, 40 s** PKV(20%) + AS	**Curing** 3 min@70 °C		
20%2L	2	**1000 rpms, 40 s** PKV(10%) + AS	**Curing** 3 min@70 °C	**1000 rpms, 40 s** PKV(10%) + AS	**Curing** 3 min@70 °C

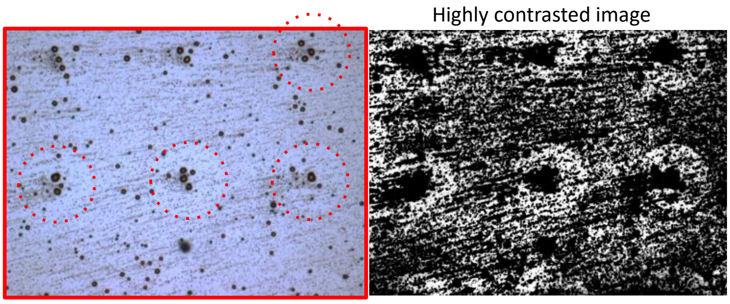

Figure S3. Optical image of sample 10%1L. (**Left**) detail of the square array of big crystallites at the centre of the GO spots. (**Right**) Same image with a high contrast, showing the emptied region around the big crystallites at the centre of each GO spot.

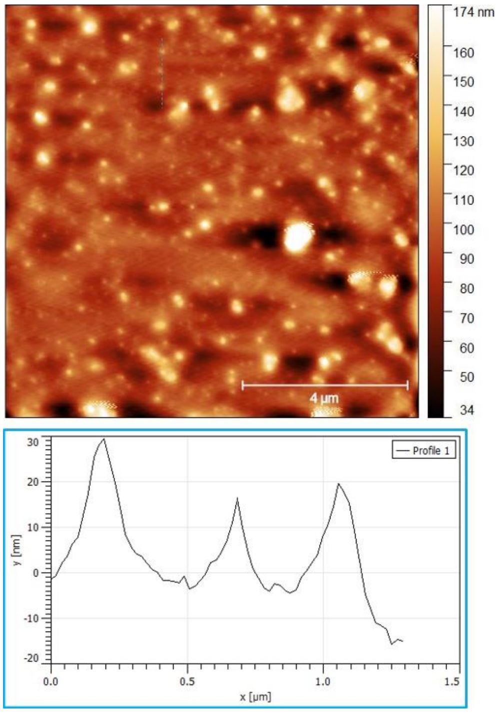

Figure S4. AFM image of sample 10%2L on top of the G region.

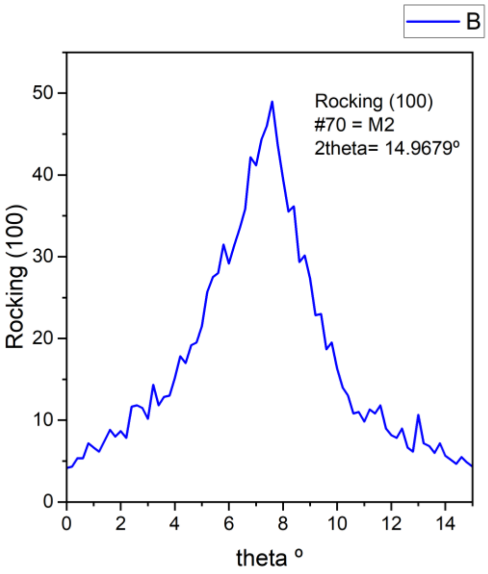

Figure S5. Rocking curve of the (100) diffraction peak of the 10%3L sample.

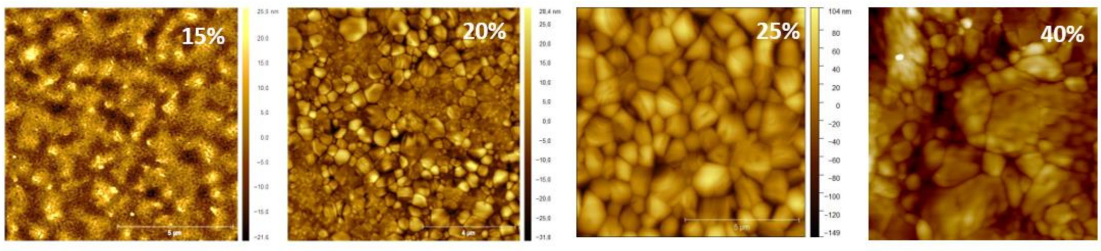

Figure S6. AFM images 10x10 microns size of 15%, 20%, 25% and 40% $MAPbBr_3$ thin films on glass.
